# Notices

**Published:** 1868-02

**Authors:** 


### NOTICES.

Rev. W. A. Scott, D.D., known as one of the best Biblical expositors in the Presbyterian Church, has issued, through Randolph, "The Centurions of the Gospel," 12mo, pp. 443, which commends itself to all classes of readers for its practicality and sterling Christian character; especially to young men about choosing a calling in life; illustrating our responsibility for our fellow-men, and the duty of prayer for our rulers and all who are in authority. Second Edition. This book deals largely with the men of our generation—Ruskin, Doctor Boardman, General Halleck, Doctor Weiglaner, General Jackson, Havelock, Sir Walter Scott, Captain Vicars, Webster, Marshal Suwarrow. Soldiers may be pious; ought there to be war? "Young men, let me beseech you to choose an occupation which will bear the scrutiny of the last day;" closing the volume with the stirring lines:

"Let us, then, be up and doing,
With a heart for any fate;
Still achieving, still pursuing,
Learn to labor and to wait."

London Lancet, republished by William C. Herald, No. 32 Beekman street, New-York. Supplied to the trade by the American News Company, Nos. 119 and 121 Nassau street, New-York. This is a journal of foreign and British medicine, physiology, surgery, chemistry, criticism, literature, and news, edited by J. H. Bennett, M.D., and T. Wakley, Jr., M.R.C.S.E., published monthly, in London. No. 12, for December, 1867, contains Murchison on diseases of the liver; Johnson on pathology of the liver and kidneys; prognosis and treatment of acute diseases from the indications of the pulse; simulated aneurism; idiocy and its relation to tuberculosis; monomania and civil law; antiseptics in practical surgery; iodine inhalation in diphtheria; tubercular meningitis; ligature of various arteries; methods and aims of medical education; force and work; diagnosis of insanity; fashion; music; children; portability of cholera, etc., etc.

Fire on the Hearth.—In our back-parlor, No. 2 West Forty-third street, New-York, may be seen, any night, the most splendid fire all around the circumference; that is to say, within the circuit of a mile or two, or a dozen, or fifty, as any reader of the Journal may see for himself, a cordial reception being warranted, if he will only tell in a minute, or rather twenty seconds, what he wants, without apology, excuse, or any thing else, for we want it to be seen and to be enjoyed. There is no fire superior to it in existence. It is a broad bed of flaming coal, two feet across, one foot deep, and two and a half feet high from the level of the floor. The hardest coal is kindled without a blower; and wood, or soft coal, or coke, or peat, can be equally well used. The fire is kindled at four o'clock in the afternoon; one scuttleful of coal is put on, and this lasts until midnight without replenishment, and without being once disturbed by a poker, consequently there is no dust. The next morning the fire is out. With a little moving of the cinders with a pointed stick, one end sharpened, the ashes fall into the cellar; the unburned cinders are sprinkled with water, which makes them more easily kindled, and are pushed to the sides; some paper, then some wood, are placed in the centre of the open grating, set on fire with a match, then the scuttle of coal is thrown on, a valve is turned to admit air from without, coming in under the grating, and the kindling goes on. When fairly burning, the outer air is shut off, and the operation is complete. By this time the children have come from school, taken their dinners, and are ready with their jokes and plays to gather around the hearthstone; the shutters are closed, the gas is brightly burning over the centre-table, now piled with the daily papers, the religious weeklies, and the magazines of the month, with such later issues from the press as may be safely placed before the eyes of the young, and, with pussy-cat in the corner, or better in the centre, basking in the genial warmth, or cosily sitting in the lap, who shall not say, "There is comfort!" and will not go at once to Dixon & Son, of Philadelphia, and order the Low-Down Grate for the happification of himself and family for the remainder of his pilgrimage?

STOVES.

But suppose, for satisfactory reasons, some reader wants a stove in a room, which will keep up a regular genial heat all day in the fiercest winter weather. Then let him order a soapstone stove, so constructed that the fire does not come in contact with the iron, except the grate itself. These stoves are open, like the Franklin, or may be made air-tight, and are the most healthful form of stove-heat ever devised in this country. Address F. A.Barton, Francestown, New-Hampshire, or 63 Monroe street, Chicago, or corner of Fourth avenue and Tenth street, New-York City, where every variety of pattern may be seen.

SCHOOLBOYS.—In the January JOURNAL, some strictures were made as to the management and errors in the public and in the private boarding and day-schools, especially of New-York City. There is a school for boys and youth where a conscientious interest is felt in the progress, gentlemanly bearing, and health of the young gentlemen connected with the institution. Small at first, but gradually growing in size as its merits became known, Professor Charlier's Institute in Twenty-fourth street near Madison avenue has become one of the very best educational establishments in the city, being principally patronized by our wealthiest families. One item is worthy of note. For some years we have been receiving orders from the Professor for individual Health Tracts. It was not, for a long time, known what use he made of them, when we learned that each pupil was provided with one or more, which he was required to take home and copy; or, if learning a modern or dead language, he was expected to translate it into such language, the object being to impress upon the mind, as indelibly as possible, the practical truths pertaining to health. And thus our tracts have passed into the classics. A few days ago, we received an order for several hundred copies of eight tracts bound together—a series for each scholar; and that the Professor's ideas of what ought to be learned by the young, as of daily practical interest in the maintenance of health, might be known, these eight tracts follow, although they have been printed more than once before. In fact, if they were carefully and studiously read every year by every reader of the JOURNAL, and if every child in every family were required to copy them, and translate them, and to commit them to memory, not one in a million would ever regret it, but, on the contrary, would feel thankful for having seen the item during all after-life. We have several times met with gentlemen, wholly unknown to us, who, having accidentally discovered us, have exhibited worn scraps of paper copied from the JOURNAL, which they had carried in their pocket-books for reference for several years. And we are quite aware of the fact that multitudes of families scattered over our broad land make it a point, in deciding what course to pursue in relation to health, to "see what HALL says." We often feel sad to think how much better others could bear these responsibilities—men of greater age, of wider experience, of more solid learning; hence, we try to be always on the safe side, and to speak from personal observation and experience. At the same time, it is a comfort to think that, in the main, men of medium capacities are, the world over, the most safely and solidly useful men. In fact, Fowler told us one day, *incog.*, supposing he were talking to John Smith, "You are pretty good in any thing—great in nothing;" and we have been mad at phrenology ever since, and abuse it every time we have a chance. The editors of the *Phrenological Journal* have, at various times, written for our portrait, or to examine our mug gratuitously; but sitting for such an object, or for a portrait, is so particularly ridiculous or stupid that we have uniformly declined the honor. Besides, what have we to hope for? Our skull is completed; we can't be great, so we think the very best thing we can do, is to subside into our shell, stay there, and let the world wag on as best it likes. Reader! did you ever know a man to sit for his photograph who didn't try to look fierce, or did you ever know a phrenologist to manipulate a numbskull, or a scoundrel, who did not skip over his peculiar deficiencies, and enlarge on the favorable traits? Every man has his price, unless divine grace has been put in his heart; and, putting these two things together, there is about one in a million who would not be a rogue, were he certain it would never be found out. The fact is, whoever does not admit John Calvin's doctrine that all men are totally depraved, does not know himself aright. We do not exactly know how soap-stone stoves should connect with total depravity; but it seems we have linked them together, and as both are good things to think about, we will let the matter stand; but will anticipate the reader's inquiry, why should we make a fire at four o'clock in the afternoon in Dixon's famous low-down grate? First, the afternoons of winter, in New-York City, are very often peculiarly damp, and raw, and chilly, leaving the body, after the work, and study, and business of the day are over, weak, and the mind depressed; and all, from the child of ten to the man of threescore, come home sad, weary, dispirited, and chilled; and under such circumstances, to enter a cosy, well-lighted room, with a rousing hot fire blazing on the hearth, throwing out its genial heat ten or fifteen feet away, gives a feeling of delightfulness which wakens up the whole man to a sense of comfort, thankfulness, and benignity, which is worth more than money to his whole family as well as to himself. And then what a blessed thing it is to the children just come from school; how the anticipation of it gives litheness to the body, elasticity to their steps, and cheeriness to the heart, on their way home, increasing at every step, as well-grounded hopes of heaven quicken the spirits of the aged as they are nearing the rest which has been prepared for the people of God. Happy, thrice happy are such! Reader, are you one of these? You ought to be.

A second reason for kindling a fire in our back-parlor at four o'clock in the afternoon is, that daylight is precious in New-York; it is the harvest-time of business and professional men; and loungers and loafers and persons of leisure would never muster up courage, of a cold day, to leave our fireside; and all our devices of pacing the floor, of answering in monosyllables, and looking at the clock would be so much ammunition thrown away; but they soon get tired of looking at a black hole in the side of the wall, called the "register," whence fumes of stifling heat, and close air, and sulphurous smell arise into your face, as from a young engine.

Third. No occupied room should be so warm from furnace or any other kind of heat, as that the door may stand open always; the halls of dwellings should be of a temperature about midway between the sitting-room and the street. All persons know the danger arising from a too sudden and great change of temperature; it has many a time put a perfectly healthy, robust man in his grave in forty-eight hours; hence children, and women, and invalids should studiously avoid such sudden changes. A room, to be comfortable as a sitting-room in cold weather, should have a thermometer, five feet from the floor, and near the door, about sixty-five degrees; often, in New-York City, it is twenty-five degrees out of doors; and to go from such a room into a temperature forty degrees lower, in a moment, must be pernicious always, often dangerous, sometimes deadly. Hence, in the daytime, when there is a great deal of running in and out, it is safer to have fewer fires burning; but when the cold night sets in, and all the family are at home, the luxury of abundant warmth and light merits our deepest gratitude to Him from whom all our comfort flows.

